# Abscisic Acid Insensitive 4 transcription factor is an important player in the response of *Arabidopsis thaliana* to two-spotted spider mite (*Tetranychus urticae*) feeding

**DOI:** 10.1007/s10493-017-0203-1

**Published:** 2017-12-05

**Authors:** Anna Barczak-Brzyżek, Małgorzata Kiełkiewicz, Magdalena Górecka, Karol Kot, Barbara Karpińska, Marcin Filipecki

**Affiliations:** 1 Warsaw University of Life Sciences - SGGW, Warsaw, Poland; 2 Centre for Plant Sciences, School of Biology, Faculty of Biological Sciences, University of Leeds, Leeds, UK

**Keywords:** Mite-pest, *abi4*, Stromules, Retrograde signalling

## Abstract

Plants growing in constantly changeable environmental conditions are compelled to evolve regulatory mechanisms to cope with biotic and abiotic stresses. Effective defence to invaders is largely connected with phytohormone regulation, resulting in the production of numerous defensive proteins and specialized metabolites. In our work, we elucidated the role of the Abscisic Acid Insensitive 4 (ABI4) transcription factor in the plant response to the two-spotted spider mite (TSSM). This polyphagous mite is one of the most destructive herbivores, which sucks mesophyll cells of numerous crop and wild plants. Compared to the wild-type (Col-0) *Arabidopsis thaliana* plants, the *abi4* mutant demonstrated increased susceptibility to TSSM, reflected as enhanced female fecundity and greater frequency of mite leaf damage after trypan blue staining. Because ABI4 is regarded as an important player in the plastid-to-nucleus retrograde signalling process, we investigated the plastid envelope membrane dynamics using stroma-associated fluorescent marker. Our results indicated a clear increase in the number of stroma-filled tubular structures deriving from the plastid membrane (stromules) in the close proximity of the site of mite leaf damage, highlighting the importance of chloroplast-derived signals in the response to TSSM feeding activity.

**Electronic supplementary material:**

The online version of this article (10.1007/s10493-017-0203-1) contains supplementary material, which is available to authorized users.

## Introduction

The two-spotted spider mite (TSSM), *Tetranychus urticae* Koch, represents one of the most destructive generalist mite herbivores. TSSM feeds on hundreds of plant species belonging to various botanical families (Migeon and Dorkeld 2006–2017). The ability of TSSM to flexibly adapt to multiple host plants resulted from innate tolerance to different plant xenobiotics (Dermauw et al. 2013; Grbić et al. 2011). As a cell-content-feeding pest, it preferably feeds from photosynthetically active mesophyll cells, sucks up the contents with its chelicerae, and simultaneously injects salivary secretions into the damaged tissues (Bensoussan et al. 2016). Gene expression and proteomic analyses of mite-infested plants revealed that TSSM triggers effective plant defence responses. These defence responses are connected with phytohormone control of metabolism including the biosynthesis of various defensive proteins and compounds (Agut et al. 2014; Díaz-Riquelme et al. 2016; Dworak et al. 2016; Martel et al. 2015; Zhurov et al. 2014).

Recent extensive studies by Martel et al. (2015) and Zhurov et al. (2014) had shown that jasmonic acid (JA) signalling is a key regulator of tomato and *Arabidopsis* defences against TSSM feeding. However, along with JA-dependent signalling, mite-herbivore responses are controlled by other hormonal networks such as salicylic acid (SA) and ethylene (ET) (Ament et al. 2004; Kant et al. 2004; Li et al. 2002, 2004; Martel et al. 2015). Abscisic acid (ABA), primarily defined as an abiotic stress, senescence, and development related hormone, appeared also as an important modulator of JA- and SA-regulated as well as ROS-mediated defences to pest infestation (Díaz-Riquelme et al. 2016; Erb et al. 2008; Vos et al. 2013) and pathogen infection (Ton et al. 2009). It is known that in tomato plants exposed to mite attack the ABA level changes both in mite-damaged and non-damaged (systemic) leaves (Gawrońska and Kiełkiewicz 1999). Although there is limited information about the molecular mechanism of ABA engagement in plant defence to mite infestation, based on the research on *Pieris rapae* caterpillars, Vos et al. (2013) postulated that ABA is a regulator of herbivore-induced resistance by activating primed JA-regulated defence responses upon secondary herbivore attack in *Arabidopsis*. This regulation especially targets the MYC2 dependent branch of JA signalling. Additionally, *Arabidopsis* ABA-deficient mutants such as *aba2* are not capable of accumulating JA and oxylipins after infection with the oomycete *Pythium irregulare* (Adie et al. 2007).

Abscisic Acid Insensitive 4 (ABI4) is an APETALA 2/ethylene-response element binding protein (AP2/EREBP)-type transcription factor involved in the regulation of metabolic and environmental signals. It is also integrated with the hormone regulatory network and participates in retrograde signalling pathways (Foyer et al. 2012). The retrograde signalling pathways control gene expression in the nucleus upon the stimuli originating from organelles. Chloroplast retrograde signals may be divided into those which are connected with plastid development or those which take part in the plant complex response to environmental stresses (León et al. 2012; Pogson et al. 2008). It was described that ABI4 is an important component of the plastid gene expression and tetrapyrrole retrograde signalling pathways, which may be executed by the regulation of many nuclear encoded chloroplast proteins (Koussevitzky et al. 2007). Furthermore, the role of ABI4 in plastid functioning was demonstrated by partially overlapping the transcriptome response of *abi4* and *gun1* (genomes uncoupled 1) mutants to the plastid translation inhibitor lincomycin treatment (Kerchev et al. 2011). Therefore, it was postulated that ABI4 takes part in transmission signals from the chloroplast to the nucleus in which GUN1 mediated retrograde signals participate. Moreover, the major roles of ABI4 and OXI1 (oxidative signal-inducible 1) in plant defence to the insect sucking pest *Myzus persicae* was proved by altered fecundity on the respective *Arabidopsis thaliana* mutants (Kerchev et al. 2013).

The involvement of ABI4 in retrograde communication evokes questions on the role and mechanism of plastid to nucleus retrograde signalling in this type of biotic stress. The exact mechanisms of chloroplast to nucleus signalling in the context of biotic stresses are still unclear. However, it was described that plants infected with pathogens as well as challenged with abiotic stresses induce the formation of stroma-filled tubular structures deriving from the plastid membrane, called stromules, which make contact with the nucleus (Brunkard et al. 2015; Caplan et al. 2015; Gray et al. 2012; Holzinger et al. 2007; Kwok and Hanson 2004; Schattat and Klösgen 2011). The same effect was also observed after the exogenous application of pro-defence signals such as hydrogen peroxide (H_2_O_2_) and SA (Caplan et al. 2015). This in turn emphasizes the role of chloroplast redox signalling pathways and ROS metabolism in local and systemic responses to light stress and pathogen infection which was shown by Kangasjärvi et al. (2014). In light of the cited evidence, stromules may act as inter-organellar paths to enable transfer of chloroplast-derived immune signals, including SA and ROS, to the nucleus (Caplan et al. 2015; Gu and Dong 2015; Hanson and Sattarzadeh 2011; VanHook 2015).

In our study, using an *A. thaliana abi4* mutant, the role of the ABI4 transcription factor in the plant response to the TSSM was examined. As evidenced by our results, TSSM feeds more intensively on the *abi4* mutant and benefits by the oviposition rate increase. TSSM activity also induced stromule formation and extension. Thus, our results for the first time indicate the engagement of retrograde signalling in plant response to TSSM attack.

## Materials and methods

### Plant materials and growth conditions

We used wild-type *A. thaliana* accession Columbia-0 (Col-0) and *A. thaliana abi4* mutants which were kindly provided by Prof. Christine Foyer (University of Leeds, UK). For stromule imaging we used transgenic *A. thaliana* Col-0 plants (having a wild-type allele of *ABI4*), constitutively expressing the chimeric protein FNR/EGFP, which consists of the chloroplast targeting transit peptide of ferredoxin-NADPH-oxidoreductase (FNR) fused to an enhanced derivative of the green fluorescent protein (EGFP) (Schattat and Klösgen 2011), kindly provided by Prof. Jaideep Mathur (University of Guelph, Canada). Plants were grown under cool-white fluorescent light (100 μmol m^−2^ s^−1^) and a long-day photoperiod (16 h/8 h) at 24 °C in controlled growth chambers. Experiments were carried out using 3.5-week-old plants.

### TSSM mass population rearing and oviposition rate assessment

The Warsaw mite strain which originated from *Sambucus nigra* plants was kept in the laboratory for at least 150 generations on bean plants (*Phasoleous vulgaris* cv. Ferrari; PNOS, Ożarów Mazowiecki, Poland) as was described in Barczak-Brzyżek et al. (2017). Bean plants were grown in a growth chamber at 16 h/8 h (day/night) photoperiod, temperature 23 ± 1 °C, and 60% relative humidity. To synchronize experimental female age, males were added at the time when female deutonymphs appeared in the stock colony, and shortly after mating young females were transferred to detached fresh bean leaves until the first eggs were laid. Five-day-old females were used for the comparison of mite fecundity on Col-0 plants and *abi4* mutants. Each experimental plant was infested with 10 adult females. Females were located in the middle of the rosette and had free choice to feed. After 24 h, eggs laid on all leaves were counted. As the initial number of females on Col-0 and *abi4* plants decreased similarly due to dispersal, the TSSM oviposition rate was expressed as the average number of eggs per female per plant per day, considering the remaining females. Results were presented for two experiments and six biological replicates.

### Leaf damage assessment caused by TSSM

Leaf damage was evaluated using the same *Arabidopsis* plants. Trypan Blue (TB) staining was performed based on Keogh et al. (1980) as was described in Barczak-Brzyżek et al. (2017). Mite-infested leaves were submerged in TB solution (0.016% TB, 8% phenol, 8% glycerol, 8% lactic acid, 65% ethanol) in a 15-mL conical polypropylene tube, placed in a boiling water bath at 95 °C for 2 min, and then left in staining solution overnight at room temperature. Later, leaves were cleared with 6 M chloral hydrate solution diluted in water (Avantor, Poland). Stained leaves were observed and digital images of the leaves were captured by a Leica M165-FC stereomicroscope (Leica Microsystems, Wetzlar, Germany). Quantification of mite-induced leaf damage was performed using ImageJ software (Schneider et al. 2012): leaf area was outlined, image was binarized and damage area was calculated.

### Stromule visualization

In the stromule visualization experiment we used freshly cut, mite-infested (10 females/24 h) and non-infested (control) *Arabidopsis* FNR/EGFP leaves. The changes in chloroplast membrane dynamics were observed in the cells in the close vicinity of the mite feeding sites under a Zeiss LSM710 confocal microscope (Carl Zeiss, Jena, Germany). GFP was excited with a 488-nm laser and emissions were detected from 500 to 530 nm. Stromule frequency was calculated on an area of 143 × 143 µm in both mite-infested and control leaves. Calculation was performed for 10 biological replicates. Stromule measurement was made using ImageJ software (Schneider et al. 2012) for 33 (control) and 93 (TSSM) observations. In the experiment, stromules longer than 4 µm were classified as ‘long’ and below 4 µm as ‘short’.

## Results and discussion

**Fig. 1:**
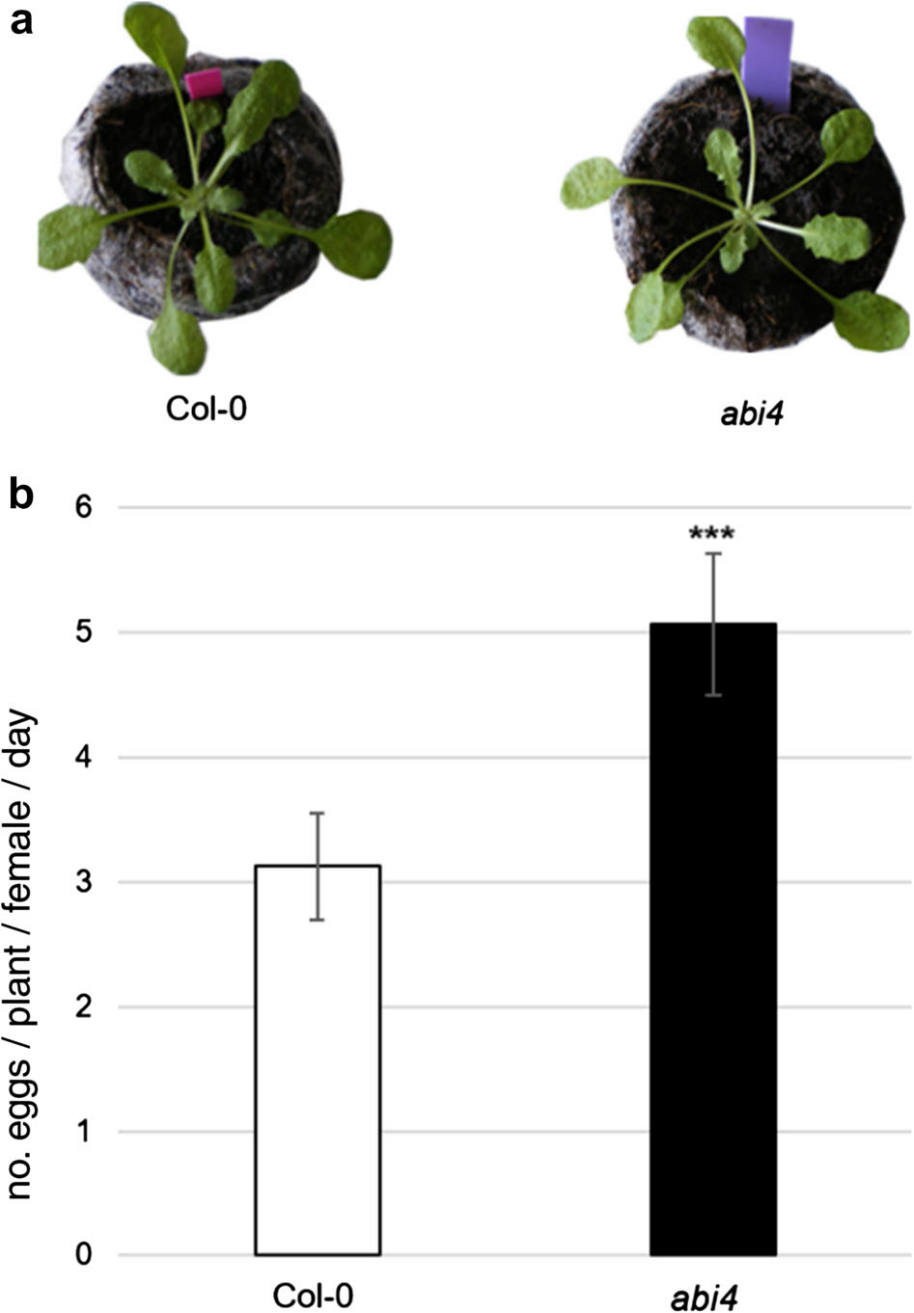
**a** Phenotype of *Arabidopsis thaliana* wild type (Col-0) and *abi4* mutant plants. **b** Mean (± SD; n = 6) TSSM oviposition rate. Asterisks represent a significant difference (two tailed Student’s t-test: *p* < 0.001)

**Fig. 2:**
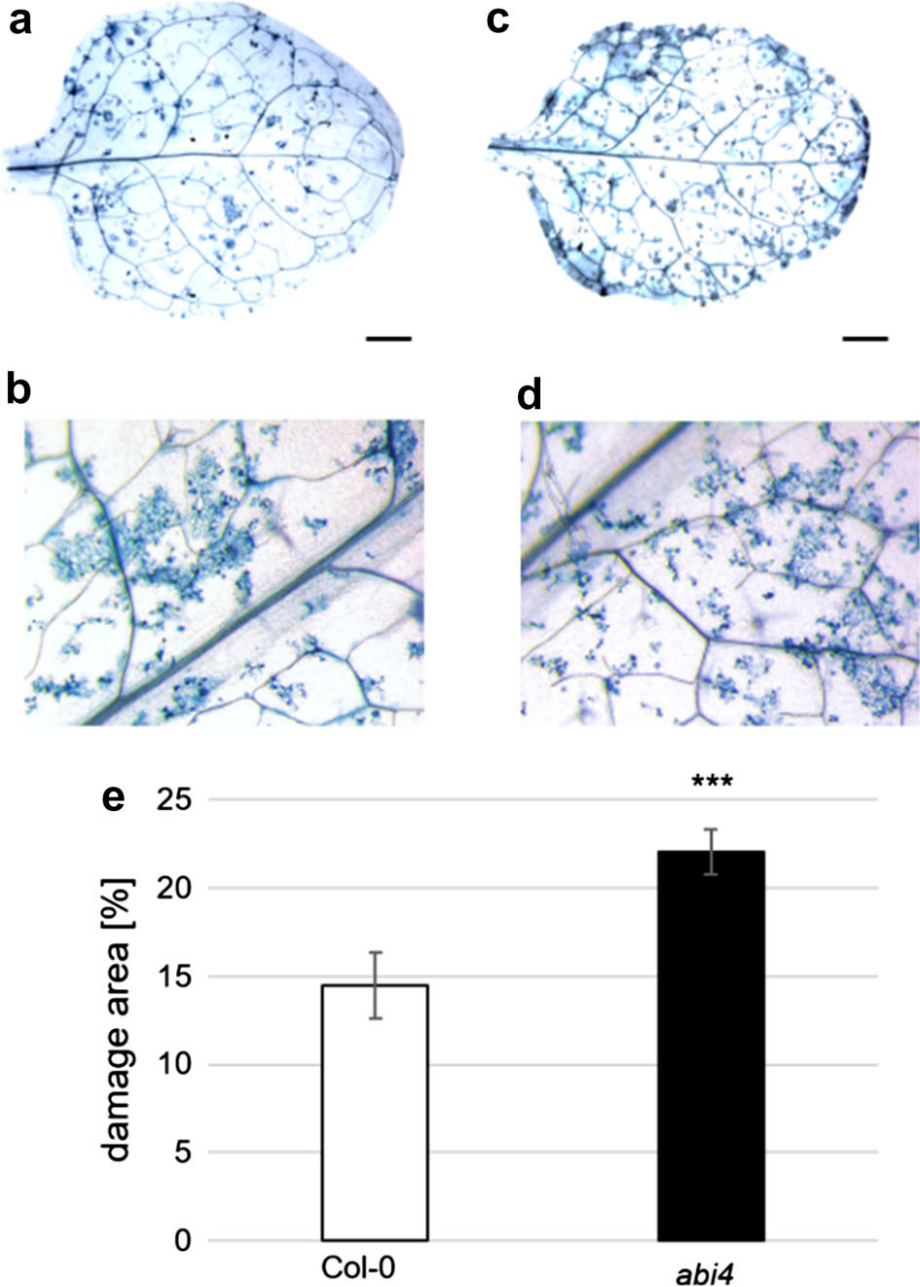
Mite-induced leaf damage symptoms visualized by TB staining 24 h post-infestation of *Arabidopsis thaliana* **a**, **b** Col-0 and **c**, **d** *abi4* mutant plants. Scale bars 2 mm. **e** Mean (± SD; n = 5) area of leaf damaged (%). Asterisks represent a significant difference (two tailed Student’s t-test: *p* < 0.001)

To investigate the role of ABI4 in the plant response to TSSM, we assessed mite female reproduction performance using the *abi4* mutants. The rate of female oviposition was significantly greater on the *abi4* mutants than on *Arabidopsis* Col-0 plants (Fig. 1), suggesting that the *abi4* mutants were more susceptible to mite attack. Furthermore, TB vital staining, which differentiates intact leaf cells and those with disrupted cell membranes (because only damaged cells accumulate the dye and appear blue), allowed identification of the mite-induced leaf damage area (Fig. 2). In our study, the damage caused by mite feeding activity was more visible in the *abi4* mutant leaves than in the wild-type (Col-0) leaves, which was also reflected in the quantification of the damage area (Fig. 2e). These observations proved that ABI4 is an important player in plant defence against this mite-pest, and confirmed that mite-associated plant defence is controlled by ABA.

**Fig. 3:**
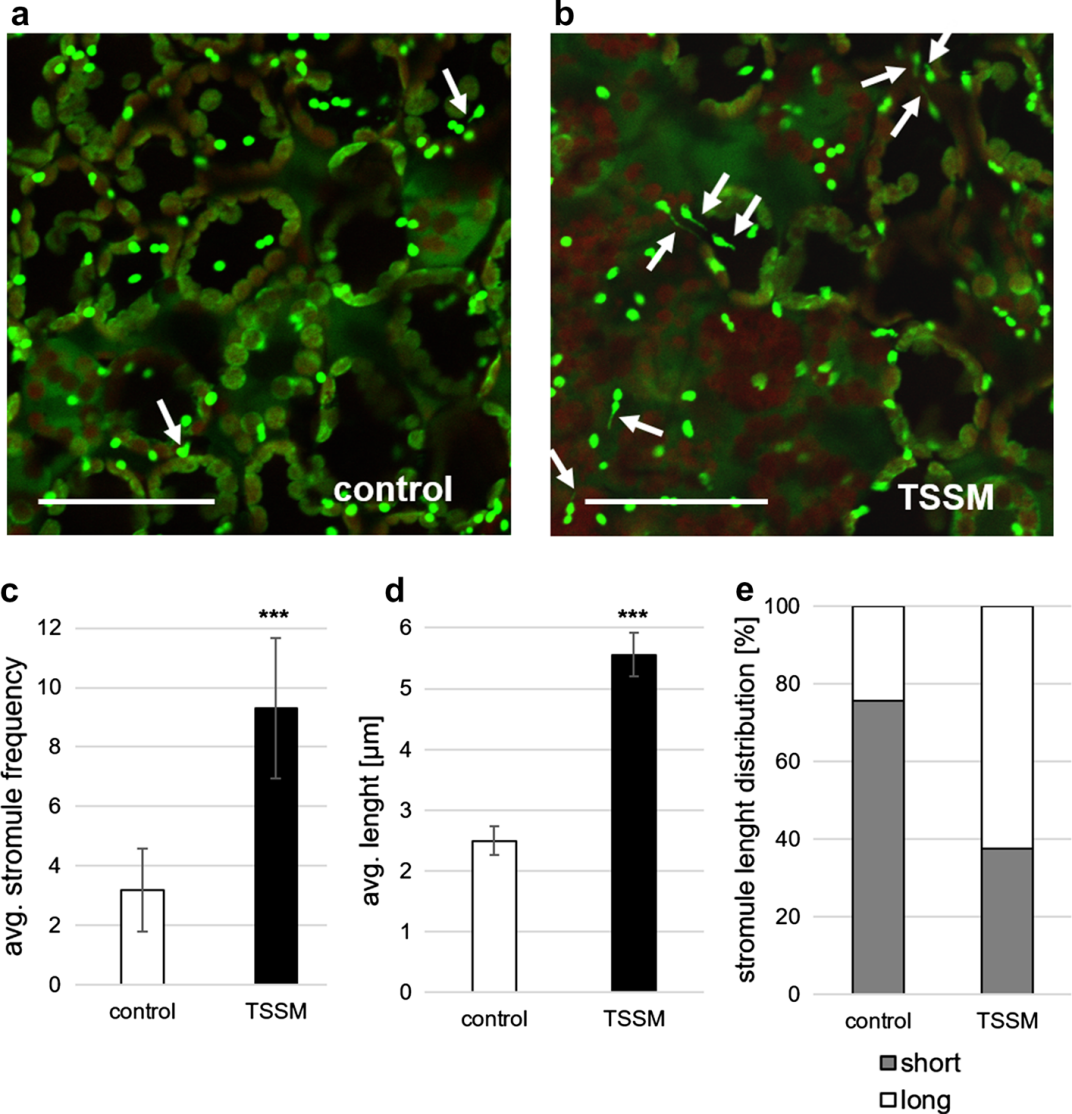
Visualization of stromule formation in **a** control and **b** TSSM infested leaves. Stromules are indicated by white arrows. Scale bars 50 µm. **c** Mean (± SD) stromule frequency and **d** mean (± SE) stromule length (μm). Asterisks represent a significant difference (two tailed Student’s t-test: *p* < 0.001). **e** Distribution of stromule lengths (%)

Additionally, TSSM feeding on plants constitutively expressing the chloroplast targeted GFP for 24 h accelerated the formation of stromules in the chloroplasts of cells in the proximity of mite-induced damage (Fig. 3, Fig. S1). We observed not only an increase in the number of ‘stroma filled tubules’ in mite-infested leaves (Fig. 3c, Fig. S1), but they were also much longer (Fig. 3d, e; Fig. S1). Such observations point out the role of stromules in plant response to mite-pest stress. Furthermore, the mite-induced abundance of stromules corresponds with the results of a previous transmission electron microscopic study, which reported that mite feeding provokes the mesophyll cell chloroplasts to form elongated cudgel-shaped protrusions and induce catechol phenolic deposition (Kielkiewicz 1999). Taking into consideration that stromules probably function in intracellular communication by exchanging signalling molecules, metabolites, or proteins (Hanson 2015; Hanson and Sattarzadeh 2013; Mathur et al. 2013; Schattat and Klösgen 2011), we may assume that this is an important retrograde or bidirectional stress information channel activated by mite infestation. Further study should establish the biological significance of mite-induced stromule formation.

It is worth noting that an increasing amount of data emphasizes that chloroplasts, which are the main source for production of immune signals in plants (e.g., SA and ROS), may directly and indirectly modulate plant defence against invaders. So far, strong evidence revealed the function of stromules during effector-triggered immunity (ETI) and programmed cell death (PCD), but the biological significance of stromule-nucleus communication in plant immunity needs to be explained (Caplan et al. 2015; Gu and Dong 2015). Additionally, non-photochemical quenching (NPQ), which is a process controlled by the trans-thylakoid proton gradient, plays an important role in the plant response to insect herbivore specialists (*Plutella xylostella*) and generalists (*Spodoptera littoralis*), which was documented in dual-choice feeding experiments with a *npq4*-*1* mutant and PsbS over-expressing plants (Johansson Jänkänpää et al. 2013).

Another example of retrograde regulation of plant defence is the plastidial signalling metabolite, 2-*C*-methyl-d-erythritol cyclopyrophosphate (MEcPP), which was able to induce JA responsive genes even in the presence of elevated SA in the *ceh1* mutant (Lemos et al. 2016). Such evidence and our results highlight an important role for chloroplasts as well as intracellular plastid-derived signals in the defence response to insect and mite pests. Moreover, they indicate that ABI4 is a crucial component of chloroplast retrograde signalling which modulates the plant response to TSSM infestation. These findings put special attention on the importance of information transfer between the plastids and nucleus, which seems to be an ancient and universal module of adaptive responses to environmental stresses.

## Electronic supplementary material

Below is the link to the electronic supplementary material.

Fig. S1 Visualization of stromule formation in control and TSSM infested leaves. Stromules classified as long (white arrows) and short (red arrows). Scale bars: 50 µm. (TIFF 1105 kb)
