# Supplementary figures and images for: Abscisic Acid Insensitive 4 transcription factor is an important player in the response of Arabidopsis thaliana to two-spotted spider mite (Tetranychus urticae) feeding
Source: Exp Appl Acarol. 2017 Dec 5;73(3):317–26. doi: 10.1007/s10493-017-0203-1 (PMC5727149; doi:10.1007/s10493-017-0203-1)

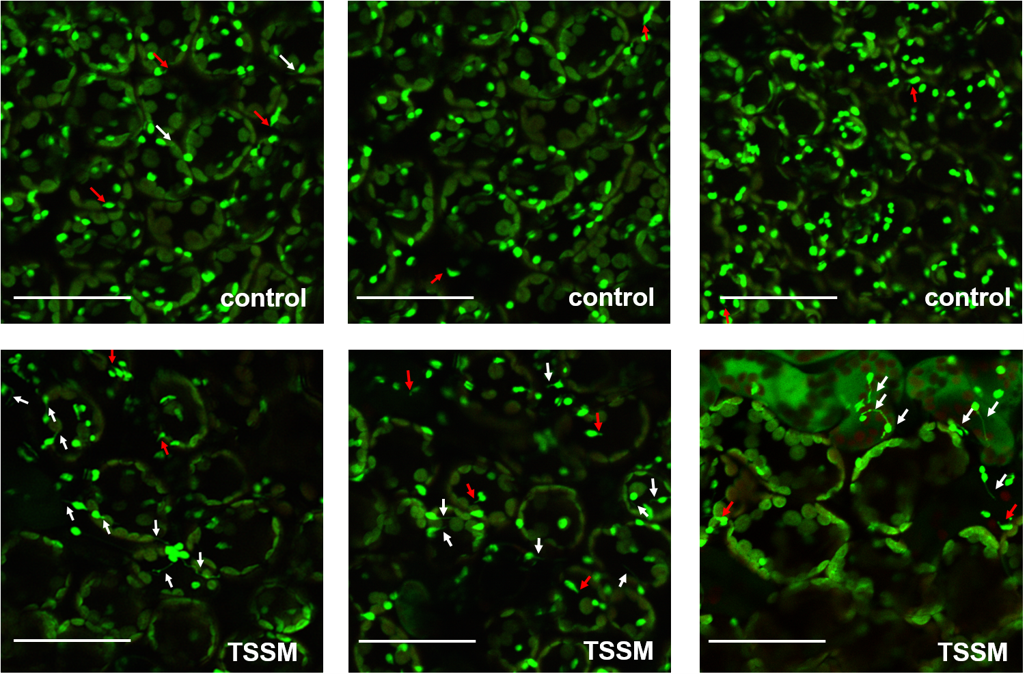

Supplement: Supplementary file 1 — Visualization of stromule formation in control and TSSM infested leaves. Stromules classified as long (white arrows) and short (red arrows). Scale bars: 50 µm. (TIFF 1105 kb) [file 10493_2017_203_MOESM1_ESM.tif]
